# Supraspinatus management in reverse total shoulder arthroplasty for proximal humerus fractures: current evidence and practical considerations

**DOI:** 10.1016/j.jsea.2026.100096

**Published:** 2026-09-21

**Authors:** Tanawan Kongmalai, Pinkawas Kongmalai

**Affiliations:** a Division of Endocrinology and Metabolism, Department of Medicine, Faculty of Medicine, Siriraj Hospital, Mahidol University, Bangkok, Thailand; b Department of Orthopedics, Faculty of Medicine, Kasetsart University, Bangkok, Thailand

**Keywords:** Reverse shoulder arthroplasty, Proximal humerus fracture, Supraspinatus, Tuberosity healing, Greater tuberosity, Fracture arthroplasty

## Abstract

**Background:**

Reverse total shoulder arthroplasty (rTSA) has become an established treatment for complex proximal humerus fractures in older adults. Although tuberosity healing remains an important determinant of post-operative rotational recovery, management of the supraspinatus tendon during fracture rTSA remains controversial. Because the supraspinatus is attached to the greater tuberosity fragment in acute fracture settings, preservation may influence fragment stability, reduction tension, and functional outcome.

**Methods:**

A focused narrative review was performed to evaluate biomechanical studies, comparative clinical investigations, and fracture-specific rTSA outcome literature addressing supraspinatus management during rTSA for proximal humerus fractures. Particular attention was given to implant design, tuberosity healing, and post-operative rotational outcomes.

**Results:**

Available biomechanical evidence suggests that preservation of the supraspinatus tendon may increase displacement of the greater tuberosity fragment, particularly in medialized prosthetic designs. Limited comparative clinical studies demonstrate no consistent functional advantage with supraspinatus preservation and, in some series, improved external rotation following tenotomy or excision. Across the broader fracture rTSA literature, tuberosity healing remains associated with improved post-operative rotation and shoulder function, suggesting that supraspinatus management should be considered within the context of overall tuberosity reconstruction strategy rather than as an isolated technical step.

**Conclusion:**

Current evidence does not support routine preservation or routine excision of the supraspinatus tendon during fracture rTSA. Instead, management should be individualized according to implant design, fracture morphology, reduction tension, and tuberosity fixation strategy, with the primary objective of optimizing tuberosity healing and post-operative rotational recovery.

Reverse total shoulder arthroplasty (rTSA) is increasingly used for displaced 3-part and 4-part proximal humerus fractures in older adults^12^^,^^14^ because it provides more predictable pain relief and forward elevation than hemiarthroplasty in appropriately selected patients, and current reviews continue to support its role as a primary treatment option in complex fracture patterns.^15^

Despite this progress, several technical details remain unresolved. Among them, management of the supraspinatus tendon during fracture rTSA is one of the least standardized. In cuff tear arthropathy, the supraspinatus is often absent or functionally irrelevant. In acute fracture rTSA, however, the tendon typically remains attached to the greater tuberosity fragment and therefore becomes part of the fracture construct itself. This makes the tendon relevant not only as a soft-tissue structure but also as a potential deforming force that may influence reduction, fixation, healing, impingement, and post-operative rotational recovery.

The problem is clinically important because tuberosity healing remains associated with improved external rotation and better patient-reported function after fracture rTSA, even in the reverse setting.^5^^,^^7^ Recent reviews continue to emphasize that union and appropriate positioning of the tuberosities contribute meaningfully to the final outcome.^9^

Recent broader reviews of fracture rTSA have addressed supraspinatus retention vs. release as one component of fracture arthroplasty.^15^ The present review narrows the scope specifically to supraspinatus management, integrating the limited direct comparative clinical evidence, fracture-specific biomechanical data, and broader literature on implant design and tuberosity reconstruction. The purpose of this review is to provide a focused synthesis of the available evidence and a practical, construct-dependent framework for intraoperative decision-making.

## Methods

This study is a focused narrative review of the available evidence regarding supraspinatus management during rTSA for acute proximal humerus fracture. Priority was given to direct comparative clinical studies, fracture-specific biomechanical analyses, and recent reviews addressing tuberosity healing, implant design, and technical factors in fracture rTSA. Additional relevant studies were identified through reference screening of key articles. Because only 2 retrospective comparative clinical studies and 1 fracture-specific biomechanical study directly address supraspinatus preservation vs. release during fracture rTSA, quantitative synthesis was not considered appropriate. Accordingly, this review was designed as a focused narrative synthesis rather than a systematic review or meta-analysis.

## Why supraspinatus management matters

The key issue is not simply whether the supraspinatus is required after rTSA. In fracture rTSA, the more relevant question is whether the tendon helps or harms the reconstructed greater tuberosity construct. The supraspinatus may preserve soft-tissue continuity and possibly contribute to compressive force sharing. Conversely, because it remains attached to the greater tuberosity fragment, it may also generate superior and medial pull that increases difficulty of reduction or promotes secondary displacement (Table I).

**Table I:** Direct comparative studies of supraspinatus management during fracture rTSA

Study	Design and cohort	Supraspinatus groups	Implant and follow-up	Main functional findings	Main radiographic findings	Key limitations
Bonnevialle et al^4^	Multicenter retrospective cohort; 150 patients	Excision: n = 117; Preservation: n = 33	Grammont design; 155° humeral inclination; non-lateralized glenosphere; mean follow-up 59 mo	External rotation was greater after excision (22° vs. 13°, *P* = .010). Constant score (61 vs. 59, *P* = .520), SSV (73% vs. 73%, *P* = .430), anterior elevation (125° vs. 128°, *P* = .450), and internal rotation (5 vs. 4 points, *P* = .200) were not significantly different.	Satisfactory greater tuberosity healing was 68% vs. 55%, favoring excision numerically but not significantly (*P* = .140).	Retrospective non-randomized design; marked group imbalance; surgeon-dependent treatment selection; variation in glenosphere size.
Siebenbürger et al^22^	Single-center retrospective comparative cohort; 159 initially treated, 76 available at the final follow-up (47.8%)	Tenotomy: n = 29; Preservation: n = 47	Aequalis Reversed FX (Grammont design); 155° humeral inclination; mean follow-up 22 ± 16 mo	Constant score (76 ± 6 vs. 65 ± 13) and normalized Constant score (92 ± 7 vs. 77 ± 16) were significantly greater after tenotomy. Significantly more patients achieved abduction >120° (89.7% vs. 21.3%) and external rotation >20° (65.5% vs. 14.9%).	No significant differences in complete tuberosity integration (44.8% vs. 44.7%, *P* = .990), resorption <50% (37.9% vs. 38.3%, *P* = .990), resorption >50% (7.0% vs. 12.8%, *P* = .700), or displacement (10.3% vs. 4.2%, *P* = .310).	Retrospective design; 47.8% final follow-up; relatively short follow-up; tenotomy adopted after a specific timepoint, introducing potential time-period bias.

*rTSA*, reverse total shoulder arthroplasty; *SSV*, Subjective Shoulder Value.

Exact *P* values for the functional comparisons in Siebenbürger et al were not provided in the original publication; these comparisons were reported as statistically significant. All other *P* values are reproduced from the original studies. No new statistical comparisons were performed in this narrative review.

This question matters because the greater tuberosity is still functionally important after fracture rTSA. In modern fracture series and reviews, tuberosity healing remains associated with better external rotation, better subjective shoulder function, and in some studies improved forward elevation. Derksen et al^6^ reported superior raw Constant score, Subjective Shoulder Value, and external rotation when tuberosities were refixated rather than excised, whereas Schmalzl et al^21^ showed that healed greater tuberosities in a 135° prosthesis were associated with improved forward flexion, external rotation, and adjusted Constant score.

Thus, supraspinatus management should be understood as one variable within a larger problem: how to achieve a stable, low-tension, near-anatomic tuberosity reconstruction that optimizes rotational recovery.

## Biomechanical rationale

The original Grammont rTSA concept was designed to restore elevation through deltoid efficiency rather than reliance on the rotator cuff. In that sense, the supraspinatus is not essential to the primary elevation mechanism after rTSA. However, this principle does not fully resolve the fracture scenario, because in acute proximal humerus fracture, the supraspinatus is attached to a healing bony fragment rather than functioning only as an isolated cuff motor. Its effect, therefore, extends beyond shoulder mechanics to fracture stability.

The most directly relevant biomechanical evidence comes from Miquel et al,^16^ who created simulated 4-part proximal humerus fractures in cadaveric shoulders treated with rTSA and compared conditions with and without the supraspinatus tendon in both medialized and lateralized designs. In the medialized model, greater tuberosity-to-diaphysis displacement and lesser tuberosity-to-diaphysis displacement were both greater when the tendon was preserved. In the lateralized model, preservation also increased greater tuberosity-to-diaphysis displacement, although the overall pattern was more complex. The authors concluded that supraspinatus excision had a positive biomechanical effect on tuberosity stability in fracture rTSA.

This study suggests 2 clinically relevant points. First, the supraspinatus can function as a destabilizing force on the reconstructed tuberosity fragment. Second, implant design likely modifies this effect. In particular, medialized constructs may be more sensitive to the deforming effect of an intact supraspinatus than lateralized designs.

Broader rTSA biomechanics support this concept. Rotator cuff preservation after rTSA does not necessarily behave as it does in the native shoulder. Prior biomechanical work has shown that cuff muscles can act as antagonists after rTSA under some conditions and can alter joint loading depending on implant geometry and residual cuff integrity. Although these studies were not fracture-specific, they reinforce the idea that preserving the supraspinatus after rTSA is not automatically beneficial and may have different consequences depending on construct design.^15^

## Direct comparative clinical evidence

Direct comparative clinical evidence regarding supraspinatus management during rTSA for acute proximal humerus fracture remains limited to 2 retrospective cohort studies^4^^,^^22^ (Table I). Despite their methodological limitations, both investigations suggest that routine preservation of the supraspinatus does not provide a clear functional or radiographic advantage over tenotomy or excision. Instead, the available data indicate that release of the supraspinatus is at least clinically acceptable and may be associated with improved post-operative rotational recovery in selected settings.

From a functional perspective, neither study demonstrated superiority of supraspinatus preservation. In the multicenter study by Bonnevialle et al,^4^ supraspinatus excision was associated with significantly better external rotation, whereas Constant score, Subjective Shoulder Value, anterior elevation, internal rotation, and complication rate were not significantly different between groups. Similarly, Siebenbürger et al^22^ reported significantly better Constant score and normalized Constant score in the tenotomy group, along with higher proportions of patients achieving clinically relevant thresholds of active abduction and external rotation. Collectively, these findings suggest that preservation of the supraspinatus does not appear necessary to optimize functional recovery after fracture rTSA and may, in some constructs, even limit post-operative motion.

Radiographic tuberosity-related outcomes have shown a less distinct pattern. Bonnevialle et al^4^ observed a numerically higher rate of satisfactory greater tuberosity healing after supraspinatus excision, although the difference was not statistically significant. Siebenbürger et al^22^ likewise found no significant between-group difference in tuberosity integration, resorption, or displacement. These observations suggest that the apparent functional benefit associated with tenotomy or excision may not be explained simply by a higher frequency of radiographic tuberosity healing. Rather, it is possible that supraspinatus release improves reduction mechanics, reduces construct tension, or facilitates more favorable soft-tissue balance without necessarily changing radiographic healing rates in a measurable way.

The current comparative evidence must, however, be interpreted with caution. Both studies were retrospective and therefore vulnerable to confounding by indication, surgeon preference, implant selection, fracture morphology, and intraoperative assessment of fragment tension. In addition, Bonnevialle et al included markedly imbalanced treatment groups, whereas Siebenbürger et al had a limited final follow-up proportion, raising concern for selection and attrition bias. Accordingly, the present literature is best interpreted as low-level comparative evidence supporting individualized, construct-dependent decision-making rather than a basis for a uniform recommendation.

Overall, the direct comparative literature does not support a clear functional or radiographic benefit for routine supraspinatus preservation during fracture rTSA. If anything, the available evidence trends in the opposite direction, suggesting that tenotomy or excision is a reasonable strategy and may improve rotational recovery in selected patients.

## Indirect clinical evidence from the broader fracture rTSA literature

Many fracture rTSA series and technical descriptions have incorporated routine supraspinatus excision or release as part of their operative strategy, usually to improve glenoid exposure, reduce superior tension on the greater tuberosity, and simplify tuberosity handling. The fact that acceptable clinical outcomes have repeatedly been reported in such series suggests that excision is technically sound and not obviously detrimental in the fracture setting. At the same time, these studies do not prove superiority of excision, because their outcomes are heavily influenced by fixation methods, implant choice, grafting, patient selection, and rehabilitation.^9^

The broader literature is more consistent on one point: tuberosity healing remains important. The recent review by Hachadorian et al emphasized continued clinical relevance of tuberosity union after fracture rTSA, and the current evidence review by Minarro and Sanchez-Sotelo^15^ likewise supports the view that healed tuberosities improve rotational function. Therefore, the question is not whether tuberosities matter but how supraspinatus management interacts with the goal of successful tuberosity healing.

This distinction is important. A surgeon may reasonably excise the supraspinatus precisely to improve tuberosity reduction and healing rather than because the tuberosity is unimportant.

## Effect modifiers

The apparent effect of supraspinatus preservation during fracture rTSA is unlikely to be constant across all cases. In practice, the tendon acts on a repair construct whose behavior is shaped by implant geometry, fixation stability, fracture morphology, and post-operative loading. For that reason, supraspinatus preservation may be mechanically acceptable in one setting yet destabilizing in another. The limited comparative literature should, therefore, be interpreted in the context of these true effect modifiers rather than as if the tendon acts independently.

### Implant design

Implant design is probably the most important modifier because it influences tuberosity position, cuff tension, distalization, lateralization, and the amount of impingement-free motion that the construct can tolerate. In the systematic review by O'Sullivan et al,^18^ which included 21 studies and 873 patients, tuberosity healing was reported to be higher with 135° implants than with 145° or 155° prostheses, whereas clinical differences across inclination groups were less consistent. Their analysis suggests that implant inclination may affect the biologic and mechanical environment of tuberosity healing even when final global function does not change in parallel.

However, the effect of lateralization is more complex and clearly system-dependent. Holschen et al^10^ found that variation in humeral inclination and glenosphere lateral offset did not lead to major differences in overall clinical outcome, although some radiographic differences such as notching and tuberosity healing patterns were observed. More recently, Bercik et al^2^ reported in a 4-part fracture cadaver model that increasing glenoid lateralization from 2 mm to 6 mm had only minimal biomechanical impact on tuberosity fracture gapping when a stem-based repair and a 135° humeral implant were used. In contrast, the randomized trial by Rius et al^20^ found that tuberosity healing with the Univers Revers system was more frequent in the 155° distalized configuration, whereas functional outcomes were similar between the 135° lateralized and 155° distalized groups at 2 years. Taken together, these findings argue against oversimplified statements such as “lateralization is always better” or “lower inclination is always superior” for tuberosity healing. Instead, implant design appears to modify the balance between healing, tension, and motion in a platform-specific manner.

### Tuberosity fixation technique

If implant design defines the background mechanics, fixation technique determines whether the tuberosities can withstand those forces, making it the second major effect modifier. Earlier pooled evidence by Onggo et al^19^ suggested that fracture-specific stems were associated with improved functional outcomes and higher tuberosity healing rates compared with standard stems, supporting the concept that fracture-adapted implants may provide a more favorable environment for tuberosity reconstruction. This is mechanically plausible because fracture-specific stems are designed to facilitate suture passage, reduce proximal bulk, improve metaphyseal support, and accommodate bone graft around the tuberosities. However, more recent comparative meta-analytic evidence has been less definitive. In a 2025 meta-analysis, Apivatgaroon et al^1^ found no significant differences between standard and fracture-specific humeral components in pain, functional scores, range of motion, tuberosity failure, scapular notching, or component loosening. Taken together, these findings suggest that stem design remains an important effect modifier, but its influence is likely mediated by the overall reconstruction strategy rather than by implant label alone. In other words, a fracture-specific stem may offer technical advantages for tuberosity repair, yet these benefits may not consistently translate into superior clinical outcomes unless they are combined with robust fixation, adequate graft support, and favorable fragment biology.

The specific fixation construct also matters. Grubhofer et al^8^ showed that cow-hitch cerclage fixation produced substantially less greater tuberosity displacement than a conventional fixation method in a fracture rTSA biomechanical model after cyclic loading. This is important because a stiffer tuberosity repair may offset at least part of the deforming effect of an intact supraspinatus. Similarly, Takayama et al^23^ reported high tuberosity healing rates using the Turned Stem Tension Band technique in complex proximal humeral fractures, and their later study suggested that the technique can maintain very high healing rates regardless of the presence of vertical sutures. The broader implication is that once fixation becomes sufficiently robust, the relative effect of supraspinatus preservation may diminish because construct stability, rather than tendon tension alone, becomes the dominant determinant of healing.

Bone grafting and cement strategy likely modify this relationship as well. In the 2024 systematic review and meta-analysis by Kaiser et al,^11^ humeral component fixation and grafting strategy were associated with differences in greater tuberosity healing, with uncemented fixation showing favorable healing patterns in pooled analysis, whereas functional outcomes were less clearly separated. More recently, Bluhm et al^3^ identified autograft from the fractured humeral head as the most significant protective factor against greater tuberosity non-union in their systematic review and meta-analysis. These findings reinforce the idea that local biology and fixation environment may be more modifiable than the tendon itself. The safety of supraspinatus preservation, therefore, likely depends less on stem label alone than on the overall quality of tuberosity reconstruction, including fixation stability, graft support, and fragment biology.

### Fracture morphology

Fracture morphology is another plausible modifier, although the evidence is more indirect. Severe comminution, head-splitting injury, poor greater tuberosity bone stock, and fracture-dislocation patterns all reduce reconstructability and narrow the margin for error during tuberosity reduction.^3^ In such settings, even a modest superior deforming force from the intact supraspinatus may become clinically relevant.

Although the studies cited here were not designed specifically to test supraspinatus management, they help frame the issue. The literature on complex fracture rTSA consistently shows that head-split and fracture-dislocation patterns are technically more demanding and often behave differently from routine 3-part or 4-part fractures.^13^^,^^17^ From a surgical standpoint, these are precisely the scenarios in which fragment reduction is more tenuous, metaphyseal support is compromised, and overtension of the greater tuberosity is least desirable. At the same time, Bluhm et al^3^ found that fracture descriptors alone were not consistently pooled predictors of non-union, suggesting that morphology should not be viewed as an isolated label but rather as a surrogate for fragment quality, repair difficulty, and the likelihood of achieving a tension-free reduction.

### Rehabilitation

Post-operative rehabilitation is an important and potentially confounding effect modifier because it determines the early loading environment experienced by the repaired tuberosities. However, rehabilitation protocols after fracture rTSA are heterogeneous, and the available evidence is insufficient to define an optimal timing of post-operative motion.

In fracture-specific rTSA, Tuphe et al^25^ compared rehabilitation initiated on post-operative day 1 with rehabilitation delayed until post-operative day 30 and reported better functional outcomes with the earlier protocol, independently of tuberosity union. Tornberg et al^24^ compared an early range-of-motion strategy, in which the sling was used primarily for comfort, with post-operative sling immobilization for 4 to 6 weeks. Early range of motion was associated with faster recovery of shoulder motion, whereas outcomes at 1 year, complication and reoperation rates, and tuberosity healing were not significantly different between groups.

These studies suggest that the timing and degree of post-operative immobilization may influence the early trajectory of functional recovery after fracture rTSA. However, because the available evidence is retrospective and the rehabilitation protocols differ substantially between studies, it remains unclear whether earlier motion provides a meaningful long-term advantage or whether its safety is equivalent across different tuberosity fixation constructs. Rehabilitation should, therefore, be individualized according to fixation stability, tuberosity quality, and the overall reconstruction rather than considered independently of the surgical construct.

## Practical surgical implications

Current evidence does not justify a universal recommendation that the supraspinatus should always be preserved or always excised. The primary intraoperative goal should remain stable, near-anatomic tuberosity reduction without overtension.

Tenotomy or excision appears most reasonable when the greater tuberosity cannot be reduced anatomically with the tendon intact, when glenoid exposure is limited, when a medialized construct is used, or when superior pull on a fragile fragment is obvious. Preservation may be considered when the greater tuberosity is reconstructible, tendon quality is acceptable, implant design is more forgiving, and reduction can be achieved without excessive tension. A practical comparison of supraspinatus preservation and tenotomy or excision is summarized in Table II.

**Table II:** Practical comparison of supraspinatus preservation and tenotomy/excision during fracture rTSA

Consideration	Supraspinatus preservation	Tenotomy/excision	Evidence and clinical interpretation
Tendon continuity	Maintains supraspinatus continuity with the greater tuberosity fragment.	Releases supraspinatus continuity from the greater tuberosity fragment.	Preservation maintains soft-tissue continuity, but a specific functional advantage after fracture rTSA has not been demonstrated.
Greater tuberosity mechanics	Transmits supraspinatus force to the greater tuberosity and may increase deforming traction and fragment displacement.	Reduces supraspinatus-related traction on the greater tuberosity and may improve construct stability.	Cadaveric biomechanical evidence demonstrated greater tuberosity displacement with preservation, particularly in a medialized construct.
Reduction and fixation	May increase reduction tension or difficulty when the fragment is fragile, comminuted, or difficult to reduce.	May facilitate a lower-tension reduction and simplify tuberosity handling and fixation.	This is primarily a biomechanical and intraoperative consideration; the clinical effect is likely construct dependent.
Glenoid exposure	Maintaining the tendon may limit exposure in selected fracture patterns.	Release may improve glenoid exposure and facilitate component implantation.	Improved exposure has been described as a technical advantage of supraspinatus release, although it has not been shown to independently improve clinical outcomes.
Tuberosity healing	No demonstrated radiographic advantage over tenotomy/excision.	No consistent increase in tuberosity healing has been demonstrated despite the theoretical reduction in deforming force.	The 2 available retrospective comparative studies found no statistically significant difference in tuberosity healing, integration, resorption, or displacement.
Post-operative function	No clear functional advantage has been demonstrated.	Associated with better external rotation in both comparative studies and with better Constant score and selected motion outcomes in 1 study.	These findings are based on low-level retrospective evidence and should not be interpreted as proof of superiority.
Factors modifying the effect	Preservation may be reasonable when stable, near-anatomic tuberosity reduction is achievable without excessive tension.	Release may be reasonable when reduction remains tense, superior traction is evident, the fragment is fragile, or glenoid exposure is limited.	The net effect is likely modified by implant design, fixation stability, fracture morphology, graft support, and rehabilitation; management should, therefore, be individualized.

*rTSA*, reverse total shoulder arthroplasty.

Direct evidence regarding supraspinatus management is limited to 1 fracture-specific biomechanical study and 2 retrospective comparative clinical studies; therefore, the proposed clinical implications should be interpreted as construct-dependent considerations rather than universal recommendations.

Relevant direct evidence: Bonnevialle et al^4^; Miquel et al^16^; Siebenbürger et al.^22^

In other words, supraspinatus management should be treated as an adjustable variable within the overall fracture reconstruction strategy rather than as a doctrinal step (Fig. 1).

**Figure 1:**
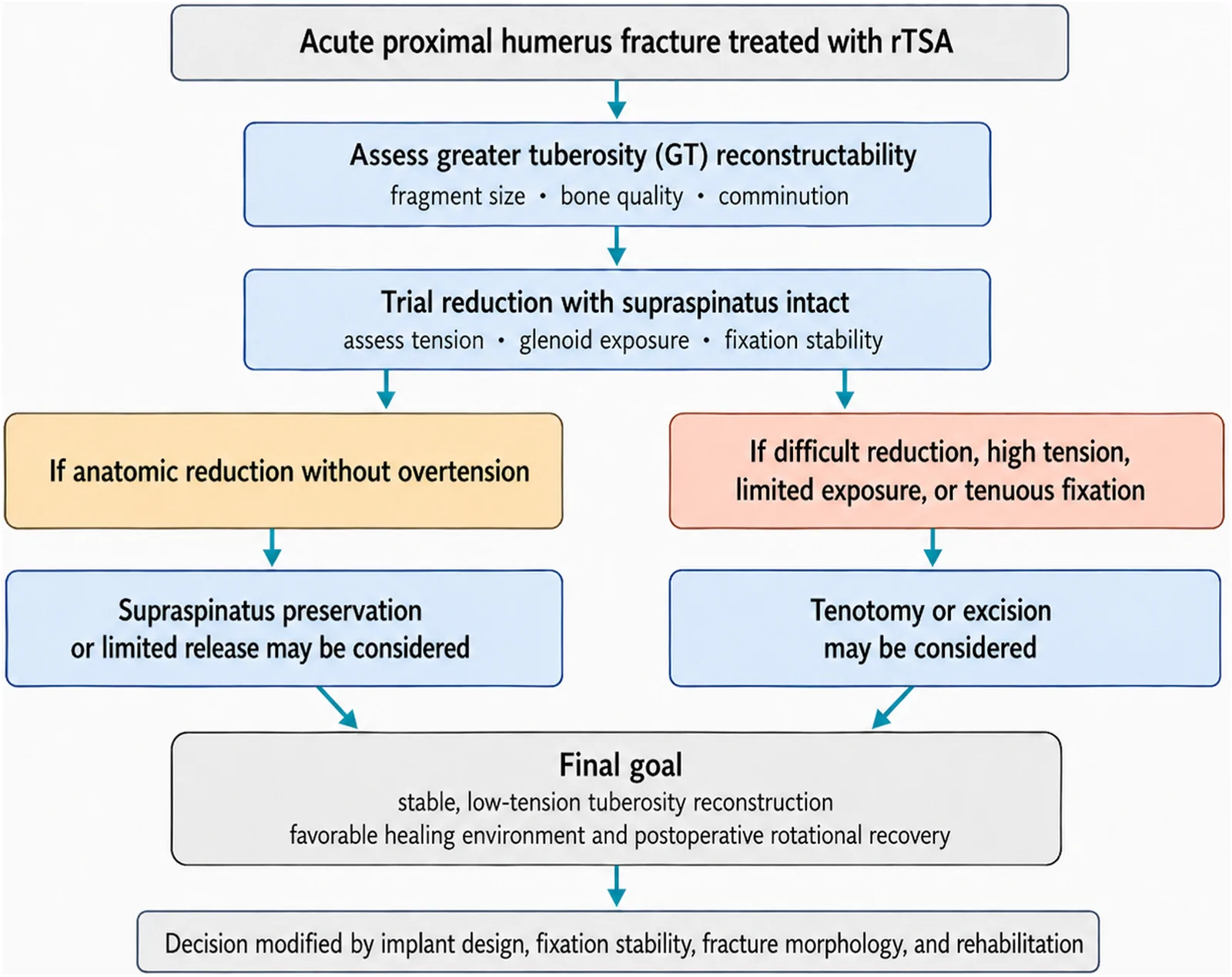
Decision-making framework for supraspinatus management during fracture rTSA. Intraoperative management of the supraspinatus during fracture rTSA should be guided by the primary goal of achieving stable, low-tension greater tuberosity reconstruction. After assessment of greater tuberosity reconstructability, a trial reduction with the supraspinatus intact can help determine whether anatomic reduction is achievable without overtension. Preservation or limited release may be considered when reduction is stable and low tension, whereas tenotomy or excision may be considered when reduction is difficult, tension is high, glenoid exposure is limited, or fixation is unlikely to be robust. The final decision remains construct dependent and may be further modified by implant design, fixation stability, fracture morphology, and rehabilitation considerations. *rTSA*, reverse total shoulder arthroplasty.

## Future directions

The principal limitation of the current evidence base is its small size, consisting of only 2 retrospective comparative clinical studies and 1 directly relevant fracture-specific biomechanical investigation. Consequently, the practical considerations proposed in this review should be interpreted as evidence-informed guidance rather than definitive treatment recommendations. Future prospective comparative studies should standardize implant systems, tuberosity fixation methods, radiographic definitions of healing, and rehabilitation protocols. Implant-specific analyses will be particularly important because the available evidence suggests that the mechanical effect of supraspinatus preservation may vary according to implant design. Future studies should also evaluate reduction quality, operative complexity, impingement, and patient-centered rotational function rather than relying primarily on global shoulder scores.

## Conclusion

Supraspinatus management during rTSA for acute proximal humerus fracture remains an unresolved but clinically relevant technical issue. The available biomechanical evidence suggests that preserving the tendon can increase tuberosity displacement, particularly in medialized constructs. The limited comparative clinical literature does not demonstrate a clear advantage for preservation and instead suggests that tenotomy or excision may provide equal or better post-operative rotation in selected settings. At the same time, the broader fracture rTSA literature confirms that tuberosity healing remains important. The most reasonable current approach is therefore individualized and construct-dependent: the surgeon should select preservation or excision according to implant design, tuberosity quality, reduction tension, and overall fixation strategy, with the over-riding aim of achieving stable tuberosity healing and functional rotation.

## Disclaimers:

Funding: No funding was disclosed by the authors.

Conflicts of interest: The authors, their immediate families, and any research foundations with which they are affiliated have not received any financial payments or other benefits from any commercial entity related to the subject of this article.

## Declaration of generative AI and AI-assisted technologies in the writing process

During the preparation of this work, the authors used ChatGPT in order to assist with language editing, readability improvement, and manuscript organization. After using this tool, the authors carefully reviewed, edited, and verified the content as needed and take full responsibility for the content of the published article.
